# Changes in Hemolymph Microbiota of Chinese Mitten Crab (*Eriocheir sinensis*) in Response to *Aeromonas hydrophila* or *Staphylococcus aureus* Infection

**DOI:** 10.3390/ani13193058

**Published:** 2023-09-29

**Authors:** Tongtong Kong, Xinyue Fan, Ngoc Tuan Tran

**Affiliations:** 1 School of Life Sciences, Qufu Normal University, Qufu 273165, China; ktt2020@qfnu.edu.cn (T.K.);; 2 Guangdong Provincial Key Laboratory of Marine Biology, Shantou University, Shantou 515063, China; 3 Institute of Marine Sciences, Shantou University, Shantou 515063, China

**Keywords:** *Eriocheir sinensis*, hemolymph microbiota, *Aeromonas hydrophila*, *Staphylococcus aureus*

## Abstract

**Simple Summary:**

Chinese mitten crab (*Eriocheir sinensis*), which has nutritional and delicious qualities, is an economically important farmed crab species. Unfortunately, bacterial diseases often occur during the breeding process in Chinese mitten crabs, resulting in significant economic losses. The role of the hemolymph microbiota in modulating physiological and biochemical functions in crustaceans is well established. However, role of the hemolymph microbiota of *E. sinensis* in relation to infections remains underinvestigated. Here, we studied the changes in the composition and function of the hemolymph microbiota in *E. sinensis* infected with either *Staphylococcus aureus* or *Aeromonas hydrophila.* Diverse patterns of structural alterations were observed in the hemolymph microbiota of crabs when exposed to various pathogens. The dynamic shifts in both the structure and function of the hemolymph microbiota provided valuable insights into the mechanisms employed by the hemolymph microbiota of *E. sinensis* in response to bacterial infections.

**Abstract:**

The Chinese mitten crab (*Eriocheir sinensis*) has significant economic potential in both the Chinese domestic and global markets. The hemolymph microbiota is known to play a critical role in regulating physiological and biochemical functions in crustaceans. However, the study of the hemolymph microbiota of *E. sinensis* in response to infections has not been undertaken. In this study, changes in the composition and function of the hemolymph microbiota in *E. sinensis* infected with either *Staphylococcus aureus* (Sa) or *Aeromonas hydrophila* (Ah) were investigated using 16S rRNA sequencing, with a phosphate buffer saline (PBS) injection serving as the control. Results showed that the dominant hemolymph microbiota of *E. sinensis* were Proteobacteria, Bacteroidota, and Firmicutes. The relative abundance of the phyla Firmicutes, Bdellovibrionota, and Myxococcota was significantly reduced in both Sa and Ah groups compared to the PBS group. At the genus level, compared to the PBS group, a significant increase in the abundance of *Flavobacterium* and *Aeromonas* was found in both Ah and Sa groups. The analysis of the functional profile showed that pathways related to ‘cell growth and death’, ‘metabolism of terpenoids and polyketides’, ‘cancers’, ‘lipid metabolism’, ‘neurodegenerative diseases’, ‘metabolism of other amino acids’, ‘xenobiotics biodegradation and metabolism’, and ‘circulatory system and endocrine system’ were predominant in the Ah group. Meanwhile, pathways related to ‘metabolism or genetic information progressing’, such as ‘translation’, ‘metabolic diseases’, and ‘cellular processes and signaling’, were enriched in the Sa group. This study revealed the effects of pathogens (*S. aureus* or *A. hydrophila*) on the maintenance of the hemolymph microbiota in *E. sinensis*. It shed light on the mechanisms employed by the hemolymph microbiota of *E. sinensis* under pathogen stimulation.

## 1. Introduction

Chinese mitten crab (*Eriocheir sinensis*) is known for its delicious flavor and nutritional content and is considered for its economic potential in both the Chinese domestic and global markets. Nonetheless, the intensive culture of *E. sinensis* has resulted in the emergence of numerous diseases caused by bacterial and viral pathogens, ultimately hindering the growth and advancement of this crab in the aquaculture industry [1,2]. Among them, bacteria are one of the main pathogens causing high mortality in cultured crabs. For example, studies proved that infections of the Gram-positive bacterium *Staphylococcus aureus* and Gram-negative bacterium *Aeromonas hydrophila* led to huge economic losses in farmed Chinese mitten crabs [3,4]. The infection of *S. aureus*, which is a widespread foodborne pathogen infecting humans and animals, can exhibit an 80% mortality rate of *E. sinensis* within 96 h [3]. Notably, the ingestion of crabs infected with *S. aureus* can also pose significant health risks to humans [5]. Furthermore, *A. hydrophila* is an emerging pathogen responsible for gastroenteritis and skin infections in humans; this is also a common pathogen causing diseases in aquatic animals, including grass carp (*Ctenopharyngodon idella*) and Chinese mitten crab [6,7]. The infection of *A. hydrophila* may result in high mortality rates in cultured animals; it exhibits wide distribution and spreads rapidly, leading to significant economic losses in the aquaculture industry [6,7]. Therefore, the study of the antibacterial immune response of *E. sinensis* is crucially important.

In recent years, there has been a growing focus on the microbiome and its complex dynamics within several host species, including humans, animals, and plants [8,9,10]. It was proven that there were various factors impacting the dynamic equilibrium of microbiota harbored the intestine of their host [11,12]. For example, the variation of gut microbiota was clearly observed to be influenced by genetics, age, sex, and medication [13]. In *E. sinensis*, numerous studies previously investigated the changes in gut microbiota under the effects of diverse environmental and biological stressors, such as heat stress, light exposure, feeding modes, salinity, and pathogen infections (such as white spot syndrome virus—WSSV) [14,15,16,17]. As an invertebrate crustacean, *E. sinensis* lacks a spine and blood vessels in its body, with all tissues and organs immersed in hemolymph [18]. This structural feature leads to a complex bacterial composition within the crab’s body. Microorganisms were previously discovered in the hemolymph of invertebrates [19]. Recent studies reported that the number of microorganisms in hemolymph can reach up to 2.6 × 10^6^ cells/mL, and these were predominantly classified in the genera *Vibrio*, *Acinetobacter*, and *Aeromonas* [20]. Previous investigations demonstrated that the hemolymph microbiota plays a crucial role in facilitating the host’s adaptation to its environment [21]. Furthermore, hemolymph serves as a vital immune tissue, displaying diverse immune functions, such as phenol oxidation, apoptosis, and phagocytosis [22,23]. Studies revealed that maintaining hemolymph microbial homeostasis is crucial for preserving host immune function [24,25]. In the case of crabs, under normal circumstances, the hemolymph microbiota is maintained in a dynamic equilibrium [20]. Nevertheless, when homeostasis is disrupted by external stimuli, it can lead to the uncontrolled proliferation of specific microbes (especially potential pathogens) harbored in the crab’s body, ultimately causing mortality in crabs [14,26]. Currently, there is substantial interest in investigating the correlation between the homeostasis of hemolymph microbiota and the regulation of antidisease immunity. However, the influence of infections (caused by *S. aureus* or *A. hydrophila*) on changes in the composition and function of hemolymph microbiota in *E. sinensis* remains poorly understood.

The advancement of sequencing technology has greatly facilitated the widespread application of 16S rRNA amplicon sequencing as the principal research method for investigating microbiota. For instance, the sequencing method has been widely applied in studying the microbiota harboring the gut of several animal species, as well as the associations between gut microbiota and diseases [27,28]. In this study, 16S rRNA sequencing was employed to investigate the characteristics of the hemolymph microbiota in *E. sinensis* after infection with either *S. aureus* or *A. hydrophila*. The results of this study demonstrated changes in the structure and function of the hemolymph microbiota in response to the stimulation of pathogens. This provides insights into the mechanisms employed by the hemolymph microbiota of *E. sinensis* in response to diverse pathogen infections.

## 2. Materials and Methods

### 2.1. Preparation of Samples

Individuals of *E. sinensis* (body weight: 50 ± 5 g) were acquired from Nanyang Lake (Jining, Shandong, China) and acclimatized for one week in tanks at a temperature of 21 °C before being challenged with 200 μL (1 × 10^6^ CFU/mL) of *A. hydrophila* or *S. aureus*. The bacterial strain *A. hydrophila* was cultured in Tryptic-Soy-Broth at 28 °C, while *S. aureus* was cultured in Luria-Betani at 37 °C until 0.6–0.8 of OD600. Then bacterial cells were collected and diluted with sterile phosphate buffer saline (PBS, Sangon Biotech, Shanghai, China). For the PBS (control) group, crabs were injected with 200 μL of PBS. Each group consisted of four replicate tanks. At 24 h postinjection, three crabs from each tank were subjected to disinfection using 75% ethanol within a sterile clean bench and used for collecting hemolymph. Subsequently, the hemolymph of crabs in the same tanks (*n* = 3) was pooled together and kept in tubes containing acid citrate dextrose anticoagulant buffer (composed of 27 mmol/L sodium citrate, 336 mmol/L NaCl (Sangon Biotech, Shanghai, China), 115 mmol/L glucose (Sangon Biotech, Shanghai, China), and 9 mmol/L EDTA (Sangon Biotech, Shanghai, China) at a pH of 7.0). Hemolymph was centrifuged at 10,000× *g* for 10 min to collect the obtained deposits, which were then stored at −80 °C for future use [29].

### 2.2. DNA Extraction, PCR Amplification, Library Preparation, and Sequencing

The CTAB (cetyltrimethylammonium bromide) method was applied for the extraction of genomic DNA from the deposits above obtained from the centrifugation of the crab’s hemolymph [30]. The concentration and purity of DNA were assessed using 1% agarose gel. The V4 region of the bacterial 16S rRNA gene was amplified using the primers 515F (5′-GTGCCAGCMGCCGCGGTAA-3′) and 806R (5′-GGACTACHVGGGTWTCTAAT-3′) in PCR reactions. Each PCR reaction performed consisted of 10 ng of DNA templates, 2 µM of forward and reverse primers, and 15 µL of Phusion^®^ High-Fidelity PCR Master Mix (New England Biolabs, Ipswich, MA, USA) [31]. Thermal cycling was carried out with an initial denaturation at 98 °C for 1 min, followed by 30 cycles of denaturation at 98 °C for 10 s, annealing at 50 °C for 30 s, and elongation at 72 °C for 30 s, with a final extension at 72 °C for 5 min. The PCR products were detected on a 2% agarose gel and purified using a Qiagen Gel Extraction Kit (Qiagen, Hilden, Germany) [31]. Sequencing libraries were generated using a TruSeq^®^ DNA PCR-Free Sample Preparation Kit (Illumina, San Diego, CA, USA), and index codes were added. To sequence the samples, the Illumina NovaSeq platform (Novogene, Beijing, China) generating 250 bp paired-end reads was applied.

### 2.3. Bioinformatics

FLASH (V1.2.7, http://ccb.jhu.edu/software/FLASH/ (8 March 2023)) was used for merging paired-end reads [32]. High-quality clean tags were obtained by quality filtering using QIIME (V1.9.1, http://qiime.org/scripts/split_libraries_fastq.html (10 March 2023)), and the chimeric sequences were detected and removed using the UCHIME algorithm (http://www.drive5.com/usearch/manual/uchime_algo.html (15 March 2023)) [33]. Operational taxonomic units (OTUs) were defined at 97% similarity using Uparse software (V7.0.1001, http://drive5.com/uparse/ (20 March 2023)) [34]. After normalizing the abundance of OTUs, the analyses of alpha and beta diversity were performed using QIIME (Version 1.9.1) and subsequently visualized using R software (V2.15.3, https://cran.r-project.org/ (26 March 2023)). The alpha diversity was analyzed based on the observed species and Shannon indexes. Principal coordinate analysis (PCoA) was used to analyze the beta diversity based on the weighted Unifrac matrix. The linear discriminant analysis (LDA) effect size (LEfSe) was performed using the Python LEfSe package (V3.7.13, https://www.python.org/downloads/release/python-3713/ (28 March 2023)) to identify differential bacterial taxa among groups. Heat map clustering was obtained using R software (V2.15.3) to present the relative abundance and function profile of microbiota in different groups. The phylogenetic investigation of communities by reconstruction of unobserved states (PICRUSt) was used to predict the function of the gut microbiota. The functional genes were predicted from the KEGG (Kyoto Encyclopedia of Genes and Genomes) with a 16S copy number [31].

### 2.4. Data Analysis

Data obtained in our research (*n* = 4 in each group) were shown as mean ± standard deviation (S.D.), which was calculated by SPSS 16.0 (IBM Corporation, Armonk, NY, USA). To assess the differences in parameters between the Sa or Ah group and the PBS (control), the *t*-test was used to analyze the differences between groups, and a significant level of *p* < 0.05 was considered. GraphPad Prism 8.0 was used to generate graphical illustrations of the data.

## 3. Results

### 3.1. General Sequencing and Microbial Diversity

**Figure 1:**
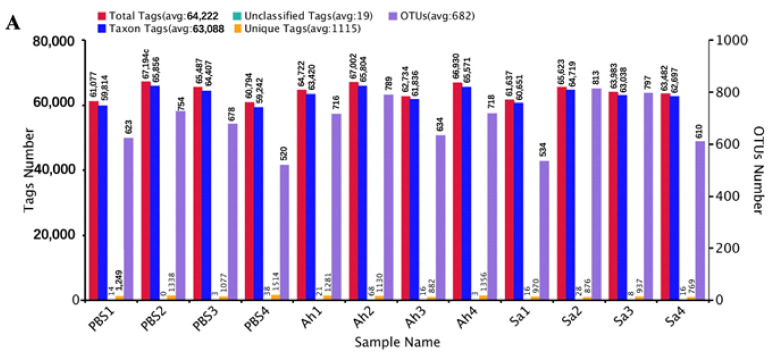

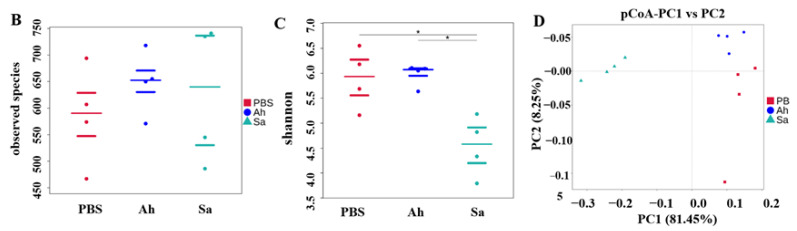
General sequencing results and microbial diversity. (**A**) Tags and OTUs numbers; (**B**,**C**) the alpha diversity of hemolymph microbiota in different experimental groups (based on the observed species and Shannon indexes, respectively). And *p* < 0.05 was marked with ‘*’. (**D**) principal coordinates analysis (PCoA), which showed the distinct separation of microbiota in different experimental groups based on the weighted Unifrac matrix.

The microbiota in the hemolymph of *E. sinensis* was subjected to 16S rRNA sequencing using the Illumina NovaSeq platform under different treatment conditions. An average of 64,222 effective tags (total tags) per sample was obtained by performing read assembly and subsequent quality control. The majority of them were taxon tags, which were then subjected to annotation. Following the clustering of total tags from each sample at 97% similarity, an average of 682 OTUs were obtained. The specific counts of tags and OTUs are presented in Figure 1A. The analysis of the alpha diversity (based on observed species and Shannon indexes) is illustrated in Figure 1B,C. Figure 1B shows the observed species counts ranging from 450 to 750, with an average of 648, 626, and 585 in the Ah, Sa, and PBS groups, respectively, but these were not significantly different among the groups (*p* > 0.05). The Shannon index revealed a significant difference between the Sa and other groups (*p* < 0.05) (Figure 1C). The results of the PCoA analysis showed that the samples in the groups formed distinct clusters, with a closer clustering between the PBS and Ah groups, indicating the dissimilarities in species complexity among the groups (Figure 1D).

### 3.2. Composition of Hemolymph Microbiota

**Figure 2:**
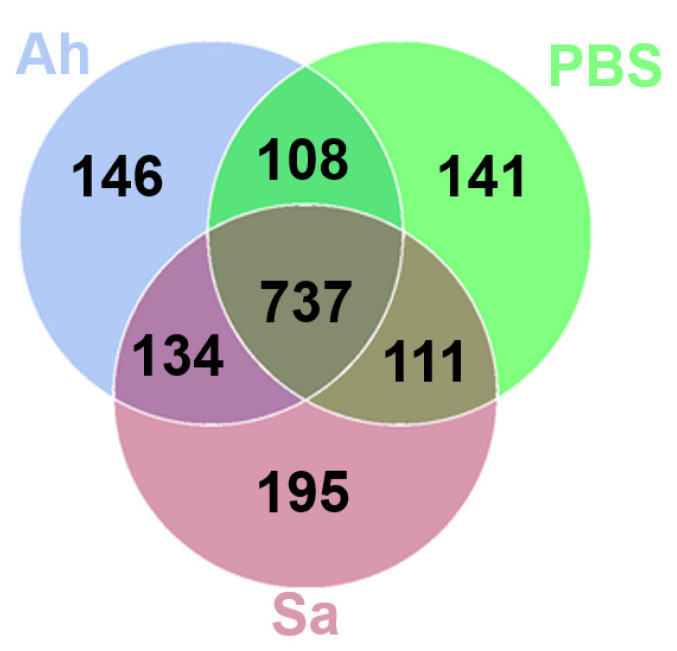
Venn diagram showing the number of unique and shared OTUs of hemolymph microbiota.

The common and unique OTUs among different groups were analyzed (Figure 2). As shown in the Venn graph, a total of 737 OTUs were detected across all samples; 111, 108, and 134 OTUs were exclusively shared by PBS and Sa, PBS and Ah, and Ah and Sa groups, respectively (Figure 2). The unique OTUs identified in the PBS, Ah, and Sa were 141, 146, and 195, respectively (Figure 2). The composition of hemolymph microbiota (at the phylum level) in different groups is shown in Table 1. The results revealed that the phyla Proteobacteria, Bacteroidota, Firmicutes, and Actinobacteriota were predominant in all groups. The relative abundance of the phylum Proteobacteria significantly decreased in the Sa group (37.65%) as compared to the PBS (69.18%) and Ah groups (68.05%) (*p* < 0.05). The abundance of Bacteroidota was significantly increased in the Ah (20.57%) and Sa (52.48%) groups when compared with the PBS group (15.12%). Conversely, a lower relative abundance of other phyla, such as Firmicutes (Ah: 2.98%, Sa: 3.21%), Bdellovibrionota (Ah: 0.18%, Sa: 0.11%), and Myxococcota (Ah: 0.26%, Sa: 0.17%), was observed as compared to the PBS group (6.40%, 0.42%, and 0.38%, respectively).

### 3.3. Intergroup Variation in the Abundance of Hemolymph Microbial Communities

**Figure 3:**
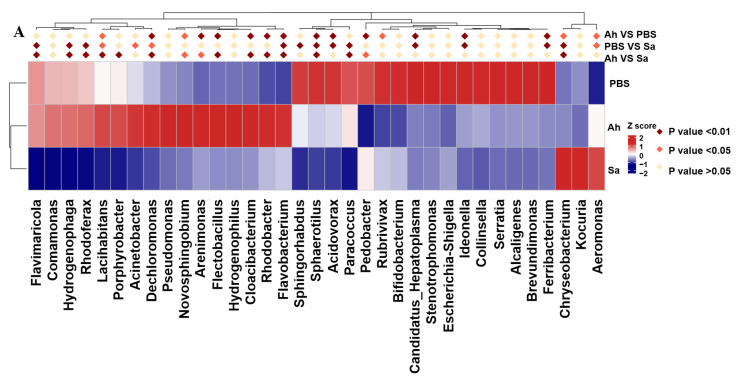

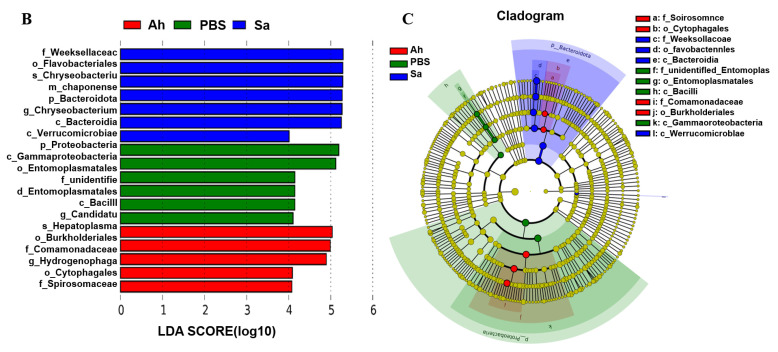
MetaStat and LEfSe analysis among different groups. (**A**) Heat map of genera with significant abundance in different groups. Z scores were obtained by subtracting the average abundance and dividing the standard deviation of all samples. Genera with *p* < 0.01, *p* < 0.05, and *p* > 0.05 are marked in brown, red, and yellow, respectively. (**B**) Histogram of linear discriminant analysis (LDA) scores for differentially abundant microbiota. (**C**) The enriched taxa from the phylum to the genus level (p: phylum; c: class; o: order; f: family; g: genus) in different groups are represented in the cladogram. Taxa enriched in PBS, Ah, and Sa groups are represented by green, red, and blue, respectively. The diameter of each circle is positively correlated with the relative abundance of the taxon.

The differences in bacterial taxa among groups based on their relative abundance were analyzed using the MetaStat and LEfSe methods. A significant increase in relative abundance of *Lacihabitans*, *Dechloromonas*, *Novosphingobium*, *Arenimonas*, *Flectobacillus*, *Cloacibacterium*, *Rhodobacter*, *Flavobacterium*, and *Aeromonas* and a decrease in *Sphaerotilus*, *Acidovorax*, *Pedobacter*, *Rubrivivax*, *Candidatus-Hepatoplasma*, *Ideonella*, *Ferribacterium*, and *Chryseobacterium* were found in the Ah group as compared to the PBS. In contrast, a higher abundance of *Flavobacterium*, *Chryseobacterium*, and *Aeromonas* and a lower abundance of *Flavimaricola*, *Hydrogenophage*, *Rhodoferax*, *Lacihabitans*, *Acinetobacter*, *Dechloromonas*, *Sphingorhabdus*, *Sphaerotilus*, *Acidovorax*, *Paracoccus*, *Candidatus Hepatoplasma*, *Ideonella*, and *Ferribacterium* were identified in the Sa group when compared to the PBS (Figure 3A).

LEfSe was employed to indicate the high-dimensional biomarkers in different groups, with the significant structure of microbiota (from phylum to genus level) in the hemolymph generated by the LDA score and cladogram (Figure 3B,C). The results showed that the microbial taxa Burkholderiales, Comamonadaceae, Cytophagales, Spirosomaceae, and *Hydrogenophage* were abundant in the Ah group, whereas the taxa Proteobacteria, Gammaproteobacteria, Entomoplasmatales, unidentified Entomoplasmatales, Bacilli, and *Candidatus Hepatoplasma* were enriched in the Sa group.

### 3.4. Changes in the Abundance of Potentially Beneficial Bacteria in Experimental Groups

**Figure 4:**
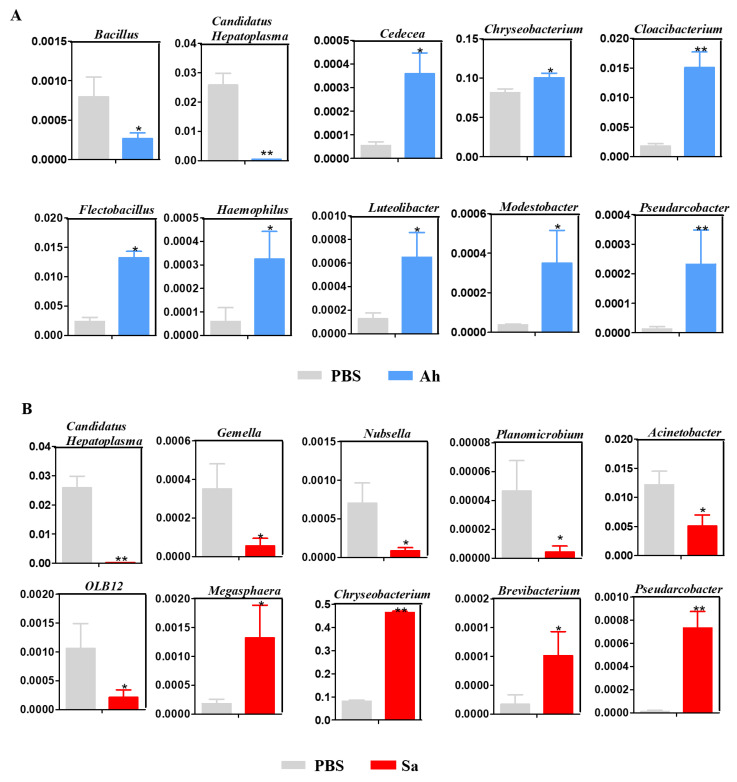
Relative abundance of hemolymph microbiota between different groups: PBS vs. Ah (**A**) and PBS vs. Sa (**B**). The *y* axis represents the relative abundance of hemolymph microbiota, while the *x* axis represents the different groups. Statistical significance is denoted by * *p* < 0.05 and ** *p* < 0.01.

To gain a deeper insight into the response of hemolymph microbiota to the invasion of exogenous pathogens, changes in the relative abundance of potentially beneficial bacteria in experimental groups were analyzed (Figure 4). As shown in Figure 4A, as compared to the PBS, the relative abundances of *Cedecea*, *Chryseobacterium*, *Cloacibacterium*, *Flectobacillus*, *Haemophilus*, *Luteolibacter*, *Modestobacter*, and *Pseudarcobacter* were significantly increased but *Bacillus* and *Candidatus Hepatoplasma* were significantly decreased in the Ah group. On the other hand, a significant increase in the abundances of *Brevibacterium*, *Chryseobacterium*, *Megasphaera*, and *Pseudarcobacter* and a significant decrease in *Candidatus Hepatoplasma*, *Planomicrobium*, OLB12, *Romboutsia*, *Gemella*, and *Nubsella* were found in the Sa group (Figure 4B).

### 3.5. Function Profile of Hemolymph Microbiota

**Figure 5:**
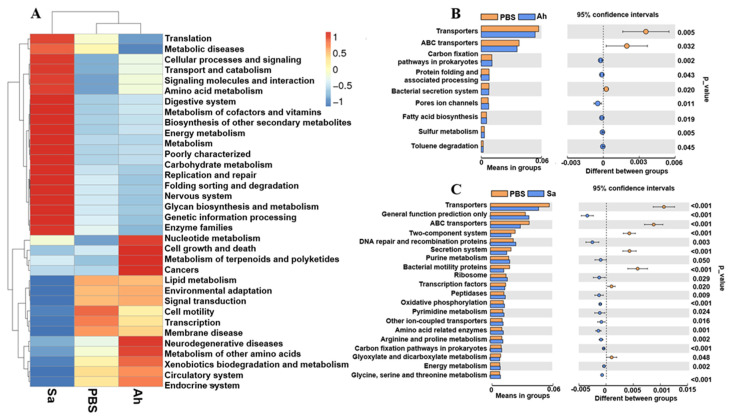
Relative abundance of the functional pathways of the hemolymph microbiota in *E. sinensis* among different groups. (**A**) The abundance of pathways at level 2. (**B**,**C**) Differences in the abundance of pathways (at level 3) between PBS and Ah and PBS and Sa, respectively.

PICRUSt was used to investigate the differences in the function profile of hemolymph microbiota in *E. sinensis* in experimental groups (Figure 5). At level 2, the microbiota in the Ah group was mainly associated with terms of ‘cell growth and death’, ‘metabolism of terpenoids and polyketides’, ‘cancers’, ‘lipid metabolism’, ‘neurodegenerative diseases’, ‘metabolism of other amino acids’, ‘xenobiotics biodegradation and metabolism’, and ‘circulatory system and endocrine system’. Meanwhile, there were some pathways related to the ‘metabolism or genetic information progressing’, such as ‘translation’, ‘amino acid metabolism’, ‘metabolic diseases’, and ‘cellular processes and signaling’ found to be enriched in the Sa group (Figure 5A).

At level 3, there were changes among the KEGG pathways in the hemolymph microbiota of *E. sinensis* in both Ah and Sa groups when compared to the PBS (Figure 5B,C). The top nine items of functional information in the hemolymph microbiota of *E. sinensis* in the Ah group were predicted (Figure 5B). The results showed a significant increase in the items of ‘carbon fixation pathways in prokaryotes’, ‘protein folding and associated processing’, ‘pore ion channels’, ‘fatty acid biosynthesis’, ‘sulfur metabolism’, and ‘toluene degradation’ and a significant decrease in the ‘transporters’, ‘ABC transporters’, and ‘bacterial secretion systems’ in the Ah group when compared to the PBS (Figure 5B). In contrast, a total of 13 functional items, including ‘general function prediction only’, ‘DNA repair and recombination proteins’, ‘purine metabolism’, ‘ribosome’, ‘peptidases’, ‘oxidative phosphorylation’, ‘pyrimidine metabolism’, ‘other ion-coupled transporters’, ‘amino acid related enzymes’, ‘arginine and proline metabolism’, ‘carbon fixation pathways in prokaryotes’, ‘energy metabolism’, and ‘glycine, serine and threonine metabolism’ were significantly increased, while seven items, including ‘transporters’, ‘ABC transporters’, ‘two-component systems’, ‘secretion systems’, ‘bacterial motility proteins’, ‘transcription factors’, and ‘glyoxylate and dicarboxylate metabolism’, were significantly decreased in the Sa group as compared to the PBS (Figure 5C).

## 4. Discussion

Hemolymph is recognized as a critical immune tissue in invertebrate crustaceans. Previous studies proved the presence of numerous microorganisms in the hemolymph of healthy invertebrates such as shrimps, crabs, and mollusks [20]. Obviously, the study on hemolymph microorganisms in Mediterranean mussel (*Mytilus galloprovincialis*) revealed a complex relationship between the innate immune system and microbes in invertebrates [35]. Moreover, investigations of the hemolymph microbiota of *Scylla paramamosain* suggested a strong correlation between the species and structure of the hemolymph microbiota and the host’s ability to resist pathogens [20]. However, there are few studies on the correlation between pathogen infection and hemolymph microbiota in *E. sinensis*. Herein, for the first time, we studied the differences in the composition and function of the hemolymph microbiota in *E. sinensis* after challenge with either *S. aureus* or *A. hydrophila*. Our results obtained a total of 195, 146, and 141 unique OTUs identified in the Sa, Ah, and PBS groups, respectively. These results indicate that the structure of the microbiota harbored in the hemolymph of *E. sinensis* was changed in response to the invasion of external pathogens, resulting in the disappearance or emergence of some bacterial taxa. This is similar to the previous study in which the structure of microbiota harbored in the gut of *S. paramamosain* was altered after a challenge with either WSSV or *A. hydrophila* [31]. This is possibly explained by the tight regulation of the host’s immunity to microorganisms, and once the composition of microorganisms is changed by infections, it can enhance the formation of a defense barrier against invading pathogens [36,37]. Thus, the changes in the hemolymph microbiota provide a basis for investigating the association between hemolymph microbes and immune regulation.

In this study, we observed that Proteobacteria, Bacteroidota, and Firmicutes were the abundant phyla in all groups, which is consistent with the previous findings that these phyla are dominant in the intestine of *E. sinensis* [15,38,39]. This is in agreement with the opinion that hemolymph microbes originate from the digestive tract in invertebrates with an open circulatory system [40]. Proteobacteria was involved in carbon complex and nitrogen removal in crustaceans, and it could play a role in stimulating the growth of the host’s immune system and supporting the maintenance of a normal immune function [38,41]. Firmicutes and Bacteroidetes were positively associated with Ca, Mg, and Cd metabolism in the gut of *E. sinensis* [42]. However, the previous study revealed that the phylum Bacteroidota (synonym Bacteroidetes) comprising Gram-negative bacteria was considered a serious threat causing diseases in the aquatic animals [41]. Here, the abundance of Bacteroidota in the hemolymph of *E. sinensis* significantly increased in both the Sa and Ah groups. This is consistent with previous findings, which indicates a significant increase in the relative abundance of Bacteroidota in the intestine of *E. sinensis* with hepatopancreas necrosis disease [43]. The phylum Firmicutes was suggested to be related to tryptophan metabolism pathways in the gut of humans and animals (such as *Apostichopus japonicus*) [44,45]. Furthermore, we observed a reduction in the abundance of Firmicutes in the hemolymph of *E. sinensis* infected with *A. hydrophila*. This is similar to the findings in a previous study, with a significant decrease in Firmicutes abundance in Tibetan sheep infected with *Echinococcus granulosus* [46]. Bdellovibrionota and Myxococcota showed a decreased abundance in response to the *S. aureus* challenge. Similarly, the previous study reported that these phyla declined in ankylosing spondylitis patients [47]. Based on our findings, we can conclude that infection with *S. aureus* or *A. hydrophila* can lead to distinct alterations in the hemolymph microbiota of *E. sinensis*. Furthermore, the members of the phylum Bacteroidota seem to be important in the response of hemolymph microbiota in *E. sinensis* to both *S. aureus* and *A. hydrophila*.

To learn more about the response of the hemolymph microbiota in *E. sinensis* to different pathogens, changes in the relative abundance of some bacteria (at the genus level) were investigated in experimental groups. Results showed that different bacteria changed at different ranges in the hemolymph of crabs under the stimulation of different pathogens. For example, *Cedecea*, *Cloacibacterium*, *Flectobacillus*, *Haemophilus*, *Luteolibacte*, and *Modestobacter* displayed a significant increase while *Bacillus* showed a decrease in response to the infection of *A. hydrophila* (Ah group). Otherwise, the abundance of *Megasphaera* and *Brevibacterium* was significantly increased, while that of *Gemella*, *Nubsella*, *Planomicrobium*, *Acinetobacter*, and OLB12 was decreased in the *E. sinensis* infected with *S. aureus* (Sa group). Interestingly, the members of the genera *Chryseobacterium* and *Pseudarcobacter* were found to be increased in both Ah and Sa groups, while the genus *Candidatus Hepatoplasma* was decreased. This is consistent with the reports in a previous study that *Candidatus Hepatoplasma* was lower in the gut of *S. paramamosain* challenged with WSSV [31]. The genus *Candidatus Hepatoplasma* was reported to involve the response of *E. sinensis* to heat stress [15]. The members of the genus *Chryseobacterium* were shown to implicate fatal nosocomial infections in humans and have antimicrobial activity in maize (*Zea mays*) against western corn rootworm larva [48,49]. Collectively, our findings indicate that the structure of the microbial community in *E. sinensis* exhibited distinct successional trajectories following infections.

Changes in the function profile of microbiota showed that the abundance of ‘metabolism of terpenoids and polyketides’ and ‘xenobiotics biodegradation and metabolism’ was significantly increased in the Ah group. It is known that the ‘metabolism of terpenoids and polyketides’ possesses antibacterial properties and contributes to the maintenance of a stable hemolymph microbiota [50]. Furthermore, the previous study reported the benefit of ‘xenobiotic biodegradation and metabolism’ in eradicating external pathogens during infections of the hepatitis B virus [51]. This suggests that the hemolymph microbiota changed its functions to the way of assisting the crabs in eliminating pathogenic bacteria during infection with *A. hydrophila*. However, in the Sa group, results showed enrichment in the abundance of pathways relating to the ‘metabolism or genetic information progressing’, such as ‘translation’, ‘metabolic diseases’, ‘cellular processes and signaling’, ‘amino acid metabolism’, ‘energy metabolism’, ‘carbohydrate metabolism’, and ‘nucleotide metabolism’, suggesting that hemolymph microbiota could supply the necessary substances and energy for the immune process and potentially trigger immune response [52,53]. This is similar to the previous findings that amino acid metabolism and energy metabolism were enriched in the carapace microbiota of lobsters (*Homarus americanus*) with epizootic shell disease [54]. The host can self-regulate to enhance their energy metabolism to adapt to changes in environmental stress [55]. Once there is failure to meet the energy demands, it can render the host more susceptible to infection by pathogens [56]. Thus, the findings in our study suggest that the hemolymph microbiota of *E. sinensis* exhibits altered metabolic activity, which could be relevant to the infections (caused by *S. aureus* or *A. hydrophila*).

## 5. Conclusions

In summary, the characteristics of the hemolymph microbiota were investigated for the first time in *E. sinensis* after challenge with either *S. aureus* or *A. hydrophila*. Distinct patterns of structural changes in the hemolymph microbiota of crabs were found under the stimulation of different pathogens. The dynamic changes in the structure and function of the hemolymph microbiota offer valuable insights into the mechanisms employed by the hemolymph microbiota of *E. sinensis* in response to infections.

## Figures and Tables

**Table 1:** Relative abundance of the top 10 bacterial phyla.

Taxonomy	PBS Group	Ah Group	Sa Group
Proteobacteria	69.18%	68.05%	37.65%
Bacteroidota	15.12%	20.57%	52.48
Firmicutes	6.40%	2.98%	3.21%
Actinobacteriota	2.38%	1.82%	1.73%
Unidentified bacteria	1.17%	1.55%	1.04%
Bdellovibrionota	0.42%	0.18%	0.11%
Gracilibacteria	0.43%	0.49%	0.21%
Desulfobacterota	0.21%	0.25%	0.18%
Acidobacteriota	0.28%	0.15%	0.12%
Myxococcota	0.38%	0.26%	0.17%
Others	4.03%	3.70%	3.10%

## Data Availability

The data presented in this study are available on request from the corresponding/first author.
